# 7^th^ Brazilian Guideline of Arterial Hypertension: Chapter 3 - Clinical and Complementary Assessment

**DOI:** 10.5935/abc.20160153

**Published:** 2016-09

**Authors:** MVB Malachias, RMS Póvoa, AR Nogueira, D Souza, LS Costa, ME Magalhães

## Clinical history and objectives

The major objectives of clinical and laboratory assessment are shown in Chart 1. Meeting those goals allows the correct AH diagnosis and prognosis, enabling choosing the better therapy for the patient.

**Chart 1:** Objectives of clinical and laboratory assessment

Confirmation of AH diagnosis by use of BP measurement
Identification of CVRF
Search for TOD, both subclinically and clinically manifested
Search for other associated diseases
Stratification of global CV risk
Assessment of evidence for suspected secondary AH

## Clinical assessment

### Clinical history

Complete clinical history with questions about time since AH diagnosis, course and previous treatment should be obtained. Information on the family history is essential to increase the chance of an accurate diagnosis of primary AH.^1^ (GR: I; LE: B). The patient should be asked about specific RF for CVD, comorbidities, socioeconomic aspects and lifestyle,^2^ in addition to previous and current use of medications or other substances that can interfere with BP measurement and/or AH treatment. Similarly, evidence of a secondary cause of AH should be investigated.

### Physical examination

Blood pressure should be measured with proper technique (Chapter 2). Anthropometric data, such as weight, height [for body mass index (BMI) calculation], abdominal circumference (AC) and heart rate (HR), should be recorded. The normal values of AC and BMI are those recommended by the International Diabetes Federation (IDF) in 2006, and can vary according to ethnicity.^3,4^ (GR: IIa; LE: C).

Assessment (Chart 2) should comprise palpation and auscultation of the heart, carotid arteries and pulses, ankle-brachial index (ABI) measurement and retinal exam.

**Chart 2:** Clinical assessment

Physical Examination
BP measurement in both arms
Weight, height, BMI and HR
Abdominal circumference
Signs of TOD
Brain: motor or sensorial deficits
Retina: lesions on retinal exam
Arteries: pulse absence, asymmetry or reduction, skin lesions and murmurs
Heart: apical beat displaced, presence of S3 or S4, murmurs, arrhythmias, peripheral edema, pulmonary rales
Suggestive signs of secondary causes*
Cushingoid characteristics
Abdominal palpation: enlarged kidneys (polycystic kidney)
Abdominal or thoracic murmurs (renovascular, coarctation of the aorta, disease of the aorta or its branches)
Decreased femoral pulses (coarctation of the aorta, disease of the aorta or its branches)
Difference of BP between arms (coarctation of the aorta and subclavian stenosis)

* For further information, see Chapter 12.

To calculate ABI, measure SBP in the arm and ankle, in both sides. An arm SBP/ankle SBP ratio greater than 0.90 is defined as normal, while PAD is defined as mild, if that ratio is 0.71-0.90, moderate, if 0.41-0.70, and severe, if 0.00-0.40.

### Basic laboratory investigation, assessment of subclinical and clinical target-organ damage

Complementary assessment is aimed at detecting subclinical or clinical TOD to better stratify CV risk. To stratify global CV risk, the classical RF (Chart 3), as well as the new ones identified, should be considered, although they have not been incorporated to the clinical scores of risk stratification.^4,5^

**Chart 3:** Additional cardiovascular risk factors

Age (men > 55 years, women > 65 years)
Smoking habit
Dyslipidemias: triglycerides > 150 mg/dL; LDL-C > 100 mg/dL; HDL-C < 40 mg/dL
DM
Family history of premature CVD: men < 55 years, women < 65 years

Of the new RF, the following stand out: fasting glycemia between 100 mg/dL and 125 mg/dL, abnormal glycated hemoglobin (HbA1c), abdominal obesity (metabolic syndrome - MS), PP (SBP-DBP) > 65 mm Hg in the elderly,^5^ history of preeclampsia, and family history of AH (for borderline hypertensive patients).

The laboratory assessment shown in Chart 4 should be part of the initial routine of all hypertensive patients.^4^

**Chart 4:** Routine tests for hypertensive patients

Urinalysis (GR: I; LE: C)
Serum potassium (GR: I; LE: C)
Fasting glycemia (GR: I; LE: C) and HbA1c (GR: I; LE: C)
Estimated glomerular filtration rate (GR: I; LE: B)
Serum creatinine (GR: I; LE: B)
Total cholesterol, HDL-C and serum triglycerides (GR: I; LE: C)*
Serum uric acid (GR: I; LE: C)
Conventional electrocardiogram (GR: I; LE: B)

* LDL-C is calculated by use of the formula: LDL-C = total cholesterol - (HDL-C + triglycerides/5) (when triglycerides < 400 mg/dL).

The Cockroft-Gault formula is used to calculate creatinine clearance:^6^ CrCl (mL/min) = [140 - age] x weight (kg) /serum creatinine (mg/dL) x 72 for men; for women, multiply the result by 0.85.

To estimate glomerular filtration rate (GFR) use the CKD-EPI equation.^7^ The interpretation of the GFR values to classify CKD (stages) is performed according to the National Kidney Foundation (NKF).^7^

The CKD-EPI equation^8^ used to estimate GFR is available at: www.nefrocalc.net

GFR (mL/min/1.73m^2^:
Stage 1: ≥ 90 = normal or high;
Stage 2: 60-89 = mildly decreased;
Stage 3a: 45-59 = mildly to moderately decreased;
Stage 3b: 30-44 = moderately to severely decreased;
Stage 4: 15-29 = severely decreased;
Stage 5: < 15 = end-stage kidney disease (KDIGO).

Certain clinical situations, discussed in Chart 5, require more detailed complementary tests.

**Chart 5:** Tests recommended for certain populations

Test/assessment	Recommended population and indication
Chest X ray	Follow-up of patients with clinical suspicion of cardiac (GR: IIa; LE: C) and/or pulmonary impairment. Assessment of hypertensive individuals with aorta impairment when echocardiogram is not available.^9^
Echocardiogram More sensitive than ECG to diagnose LVH. Important in the assessment of the geometrical forms of left atrial hypertrophy and size, analysis of systolic and diastolic function. Consider LVH when left ventricular mass corrected for body surface is equal to or greater than 116 g/m^2^ for men and 96 g/m^2^ for women.^10^	Evidence of LVH on ECG or patients with clinical suspicion of HF (GR: I; LE: C).
Albuminuria Predicts fatal and non-fatal CV events. Normal values < 30 mg/24h (GR: I; LE: C).^7,11^*	Diabetic hypertensive patients, with MS or at least two RF.
Carotid US The carotid IMT and/or identification of plaques predict the occurrence of stroke and MI independently of other CVRF. IMT values > 0.9 mm, as well as the presence of atherosclerotic plaques, have been considered abnormal (GR: IIa; LE: B).^12^	Carotid murmur, CbVD signs or atherosclerotic disease in other sites.
Renal US or with Doppler	Patients with abdominal masses or abdominal murmur (GR: IIa; LE: B).^13^
HbA1c	- When fasting glycemia > 99 mg/dL - Family history of type 2 DM or previous diagnosis of type 2 DM and obesity (GR: IIa; LE: B).^14^
Exercise test	- Suspicion of stable CAD, DM or family antecedent of CAD in patients with controlled BP (GR: IIa; LE: C).^15^
ABPM/HBPM	- According to the conventional indication of those methods (GR: IIa; LE: B).
PWV “Standard” for assessing arterial stiffness. Values greater than 12 m/s are abnormal (GR: IIa; LE: B).^16^	- Intermediate-to-high-risk hypertensive patients.
MRI of the brain: to detect silent infarctions and micro hemorrhages (GR: IIa; LE: C).^17^	- Patients with cognitive disorders and dementia.

LVH: left ventricular hypertrophy; CV: cardiovascular; RF: risk factor; US: ultrasonography; IMT: intima-media thickness; MS: metabolic syndrome; MI: myocardial infarction; CVRF: cardiovascular risk factor; CbVD: cerebrovascular disease; HbA1c: glycated hemoglobin; DM: diabetes mellitus; CAD: coronary arterial disease; ABPM: ambulatory blood pressure monitoring; HBPM: home blood pressure monitoring; PWV: pulse wave velocity; MRI: magnetic resonance imaging.

* Next figure shows the current classification and nomenclature for albuminuria and GFR according to KDIGO, 2012.^7^

## Figures and Tables

**Figure 1:**
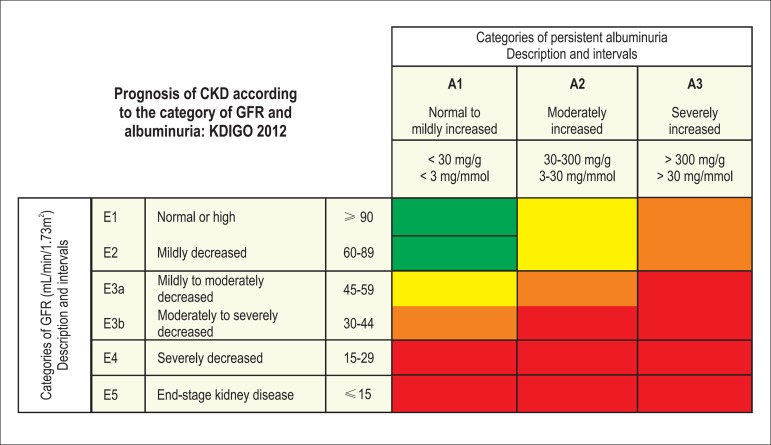
Prognosis of CKD according to the category of GFR and albuminuria. Green: low risk; yellow: moderately increased risk; orange: high risk; red: extremely high risk.
